# The market dynamics of selective serotonin re-uptake inhibitors: a private sector study in South Africa

**DOI:** 10.4314/ahs.v17i4.29

**Published:** 2017-12

**Authors:** Frasia Oosthuizen, Pariksha Jolene Kondiah, Hawa Bibi Moosa, Siddiqa Naroth, Nabeel Ismail Patel, Divashnee Reddy, Amanda Soobramoney

**Affiliations:** University of KwaZulu-Natal, Pharmaceutical Sciences

**Keywords:** Selective serotonin, private sector, South Africa

## Abstract

**Objective:**

The objective of this study was to analyse the market share of generic vs originator selective serotonin re-uptake inhibitors (SSRIs), and also compare market share of different SSRIs in the private health care sector in South Africa, over a period of 4 years.

**Methodology:**

This was a retrospective, descriptive study that measured generic market volume as a percentage of the total private SSRI market volume. Retail private sector sales data for six SSRIs available in the private sector in South Africa was evaluated. Sales data were obtained from various stages in the pharmaceutical supply chain, June 2009 – May 2013.

**Results:**

Generics constituted 86% and originators 14% of the private sector market volume of SSRIs. The share of the market volume of generic medicines increased by 29.93% over this 4-year period, while an overall increase of 27.86% in the ratio of generics to originators was observed.

**Conclusion:**

In line with policies, generic SSRIs hold a larger volume of the market in the private sector in South Africa.

## Introduction

Depression is a debilitating disorder in which patients have a low self-esteem and feel helpless, sad, and guilty.^1^ According to the World Health Organisation, depression is affecting about 350 million people worldwide, therefore the demand for curbing and treating this mental health disorder is a global challenge. A recent South African study reported that depression was the most common diagnosis, being present in nearly two thirds (63.9%) of non-fatal suicidal patients seen in a large academic hospital.^1^

Depression is amongst the most commonly occurring chronic illnesses worldwide, and cost of illness research has shown that depression is associated with an enormous economic burden.^2^ In an attempt to curb high healthcare costs, governments have placed increasing importance on the provision of low-cost, quality assured medicines.^3^ The use of generics is therefore often promoted in both the public and private sectors to reduce medicine costs, and increase product availability and consumer access.^4^ The National Drug Policy for South Africa (1996) recommends the use of generics as a means of reducing drug costs and expenditure. This contributes to a comprehensive system of procurement, distribution, drug information and rational use at all levels of the health care system in South Africa.

According to the FDA, generic medicine is identical, or bioequivalent, to an originator in terms of dosage form, safety, strength, route of administration, quality, performance characteristics and intended use. The use of generic pharmaceuticals is most frequent in industrialized countries, where prices for pharmaceuticals are usually high.^5^ Market share of generic medicines have steadily increased worldwide: from 42% in 2005 to 49% in 2009 in Europe, and an increase from 19% in the USA between 1984 and 2005.^3^

According to Kaplan, Wirtz & Stephens^3^ comparatively little is known about the private pharmaceutical market in middle-income countries, and even less is known about market dynamics between originator and generic versions of the same product. Uptake of generic medicines may be sub-optimal;^4^ barriers to the use of generic medicines include lack of incentives for physicians to prescribe generics, economic disincentives for pharmacists to dispense generics, and lack of confidence in the quality of generic medicines on the part of the patients and health professionals.^4^

Selective serotonin re-uptake inhibitors (SSRIs) selectively and powerfully inhibit serotonin re-uptake and result in a potentiation of serotonergic neurotransmission thus exhibiting therapeutic activity in depression, as well as anxiety, obsessional and impulse control disorders.^6^

This study therefore aimed to determine the trends in market volume of generic vs originator SSRIs in the private sector in South Africa, during the period June 2009 – May 2013, also comparing market share of different SSRIs during this period.

## Methodology

This was a retrospective study based on private sector sales data obtained from the IMS Health (Proprietary) Limited (South Africa) information service(s) from April 2009 to June 2013. IMS provides a useful database to the pharmaceutical and health care industries used by government, academics, drug plan administrators and pharmaceutical companies.

Volume data represents purchase by the supply chain rather than actual consumption. Data for the period June 2009 to May 2013 was analysed for this study.

The SSRIs included in the study were fluoxetine, fluvoxamine, paroxetine, sertraline, citalopram and estcitalopram; these SSRIs (both originator products as well as generics) are currently available in the private sector in South Africa.

The total generic market share was determined according to the method of Kaplan, Wirtz & Stephens^3^: the percentage of total private sector sales volume of generic SSRIs divided by the total SSRIs private sector sales volume (originator + generic) for the period June 2009 –May 2013.

For the purpose of this study a generic is identified as a pharmaceutical product intended to be interchangeable with the originator brand products, manufactured without a license from the originator manufacturer and marketed after expiry of the patent or other exclusivity rights.^4^

This study also measured market volume of different SSRIs by measuring the percentage of total private sector sales for a specific SSRI (originator + generic) divided by the total SSRIs private sector sales volume (originator + generic) for the period June 2009 – May 2013.

## Results

**Fig 1:**
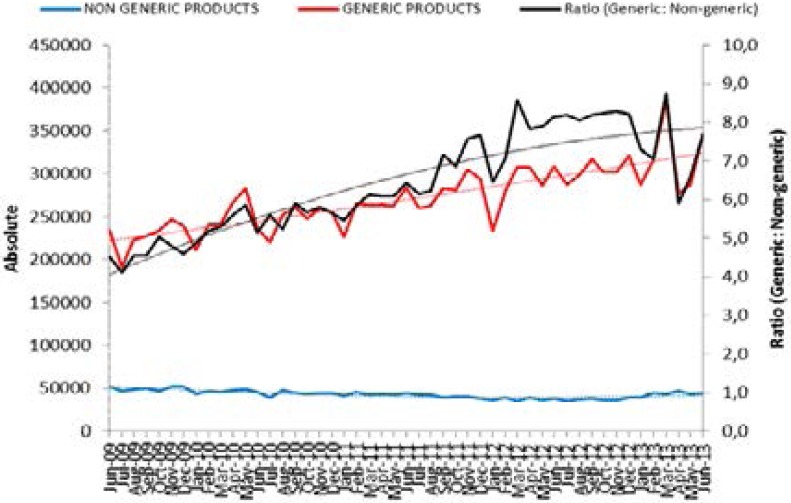
Comparison between the market volume of generics vs. originators for the period June 2009 – May 2013.

The share of the market volume of generic medicines increased by 29.93% over this 45-year period, while an overall increase of 27.86% in the ratio of generics to originators was observed. The market volume of originator SSRIs remained relatively constant during this period (Figure 1).

The market volume for different SSRIs available in South Africa over the 45-year period of the study is described in Table 1.

**Table 1:** Market volume of SSRI's for the period June 2009 – May 2013.

Fluoxetine	Comprises 29% of total SSRI market volume for period June 2009 – May 2013		
Prozac® (originator)	2% of market volume of fluoxetine	*Nuzak*® (generic product of fluoxetine with highest market volume)	45% of market volume of fluoxetine
Sertraline	Comprises 9% of total SSRI market volume for period June 2009 – May 2013		
*Zoloft*® (originator)	7% of market volume of sertraline	*Serdep*® (generic product of sertraline with highest market volume)	40% of market volume of sertraline
Paroxetine	Comprises 8% of total SSRI market volume for period June 2009 – May 2013		
*Aropax*® (originator)	26% of market volume of paroxetine	*Paxil*® (generic product of paroxetine with highest market volume)	37% of market volume of paroxetine
Fluvoxamine	Comprises 1% of total SSRI market volume for period June 2009 – May 2013		
*Luvox*® (originator)	30% of market volume of fluvoxamine	*Faverin*® (generic product of fluvoxamine with highest market volume)	63% of market volume of fluvoxamine
Citalopram	Comprises 31% of total SSRI market volume for period June 2009 – May 2013		
*Cipramil*® (originator)	4% of market volume of citalopram	*Cilift*® (generic product of citalopram with higest market volume)	73% of market volume of citalopram
Estcitalopram	Comprises 22% of total SSRI market volume for period June 2009 – May 2013		
*Cipralex*® (originator)	34% of market volume of estcitalopram	*Lexamil*® (generic product of estcitalopram with highest market volume)	50% of market volume of estcitalopram

Fluoxetine, the first marketed SSRI, still holds a substantial portion of the market volume (29%). As to be expected, the generic product Nuzak®, priced at R35.74 / unit, has a much larger portion of this market volume (45%) when compared to the originator, Prozac® (2%), priced at R427.12 / unit.

Citalopram and its enantiomer, estcitalopram, hold the majority of the remaining market volume – citalopram accounts for 31% of the market volume and estcitalopram for 22%. Once again, the generic products have a larger portion of the market volume with price most probably the most significant factor. There is a substantial price difference between the originator and top-selling generic citalopram products - Cipramil® (originator) is priced at R297.79 / unit vs R69.47 for a Cilift® (generic) unit; as well as the estcitalopram products - Cipralex® (originator) is priced at R426.34 / unit vs R175 for a Lexamil ® (generic) unit.

## Discussion

The cost savings of increased use of generic medicines can be substantial.^3^ Potentially, it is possible to improve cost-effective medicine use in the private sector if originator brands were switched to the lowest-priced generic equivalents available.^7^ Results from this study show an increase in the market volume of generic SSRIs, while there is no significant change to the market volume of originator SSRIs.

Although it should not be assumed that, if market share of an originator has decreased, the counterpart generic has increased, a previous study conducted in South Africa found a higher number of originators were replaced by their counterpart generic products.^3^ In low to middle income countries, originators that have come off patent generally cost substantially more than do their generic equivalents^7^— this is also obvious in the prices of SSRIs in South Africa as indicated above.

Despite the sharing of the same principal mechanism of action, SSRIs are structurally diverse with clear variations in their pharmacodynamics and pharmacokinetic profiles. These pharmacological and pharmacokinetic differences underly the increasingly apparent important differences amongst the SSRIs.^6^ One of the most important differences between the SSRIs is their potential to cause drugdrug interactions through inhibition of cytochrome-P450 isoforms. Knowledge regarding the CYP-isoforms involved in the metabolism of these drugs may help anticipate and avoid potentially dangerous drug-drug interactions. 8 These clinical important differences amongst the various SSRIs will impact on prescribing patterns and thus market volume of the different products.

Fluoxetine, citalopram and estcitalopram claim the largest share of the SSRI market volume in South Africa. Although pharmacologically quite similar, there are subtle differences between the various SSRIs that might influence choice.

Fluoxetine, the first SSRI launched on the market, still remains one of the SSRIs with the highest market share in the private sector in South Africa despite some disadvantages, including high drug interaction potential; common adverse effects such as headache, diarrhoea and anxiety; long half-life; and slow onset of action.^9^

Citalopram and its enantiomer, estcitalopram, are the newest SSRIs available and together share the largest volume of the SSRI market in the private sector in South Africa. Citalopram, with a slightly larger share of the market volume compared to estcitalopram, might be preferred by both prescribers and patients as it is selective and it is associated with decreased occurrence of withdrawel symptoms.^9^ Estcitalopram is the most selective SSRI and has few adverse effects compared to other SSRIs; it has a rapid onset of action, potent anxiolytic effects and is best tolerated.^9^

The remaining SSRIs claim a small portion of the market volume, which might be due to small but significant pharmacological differences. Sertraline has a low drug interaction potential, however it has many adverse effects such as nausea, insomnia, diarrhoea and headache.^9^ Paroxetine has a moderate to high drug interaction potential and it has a highly variable half-life. Paroxetine is the most potent SSRI, but least selective for serotonin re-uptake receptors.^9^ It's adverse effects include sexual dysfunction, weight gain and anti-cholinergic effects such as dry mouth and constipation. Paroxetine has a risk of discontinuation syndrome — it has withdrawal like symptoms such as nausea, headache, and flu-like symptoms, thus contributing to its low percentage of consumption.^9^ Fluvoxamine is the SSRI with the lowest market share in South Africa. This drug is mainly used for obsessive-compulsive disorder. It has a risk of discontinuation syndrome and it is the most sedating SSRI.^9^

Lastly, a large portion of the private sector are covered by medical aid. Many medical aids use a maximum medical aid price (MMAP) system for drugs that are no longer controlled by a patent and are therefore available from many sources. Patients who wish to receive a branded version that costs more than the MMAP have to pay the difference in price themselves (an example of a patient co-payment). In these cases, patients may opt for the generic product, covered by the medical aid, thus contributing to the difference in market volume between originator and generic products.

## Conclusion

Generic SSRIs hold a larger volume of the market in the private sector in South Africa when compared to originator products. This is in line with policies, even within the private health sector, to promote the use of generics and decrease pharmaceutical expenditure.

When it comes to choice between different SSRIs, these are based on price, prescriber and patient preference and pharmacological differences between the different SSRIs.
